# The role of smoking in bladder cancer risk: Unveiling racial and ethnic disparities in US and global populations, a secondary dataset analysis

**DOI:** 10.18332/tid/214106

**Published:** 2025-12-16

**Authors:** Bowen Yang, Jialin Yuan, Wenyuan Song, Hanyu Wang, Han Wang, Shufang Hou

**Affiliations:** 1 Department of Oncology, Dongguan Hospital of Guangzhou University of Chinese Medicine, Dongguan, China; 2 Clinical Medical College, Chengdu University of Traditional Chinese Medicine, Chengdu, China

**Keywords:** bladder cancer, smoking, Global Burden of Disease, tobacco control, public health

## Abstract

**INTRODUCTION:**

Bladder cancer is a common malignancy of the urinary system, with smoking recognized as its most significant modifiable risk factor. Although substantial epidemiological evidence has established an association between smoking and bladder cancer, there remains a gap in understanding the complex burden and risk patterns of bladder cancer across different populations. This study aims to investigate the potential relationship between smoking and bladder cancer risk among individuals aged ≥20 years by leveraging large-scale, multinational data.

**METHODS:**

This cross-sectional study utilized two databases: the Global Burden of Disease (GBD) 2021 and the National Health and Nutrition Examination Survey (NHANES) cross-sectional data cycles from 1999 to 2023. After excluding participants with missing data on smoking history, bladder cancer history, and other relevant variables, individuals aged ≥20 years were included. The GBD data provided macro-level estimates of bladder cancer mortality and disability-adjusted life years (DALYs) attributable to smoking both globally and within the United States. Individual-level data from NHANES were used to assess the association between smoking history and bladder cancer risk through multivariable logistic regression models, adjusting for multiple confounding factors.

**RESULTS:**

Smoking is a major risk factor for bladder cancer both globally and in the US, with the smoking-attributable burden of bladder cancer markedly higher in the US. In 2021, the age-standardized death rate (ASDR) in the US was 1.97 (95% uncertainty interval, UI: 1.57–2.47), significantly exceeding the global rate of 1.12 (95% UI: 0.94–1.35). Similarly, the US age-standardized disability-adjusted life year rate (ASDAR) was substantially higher than the global average, at 42.60 (95% UI: 34.89–51.57) versus 23.56 (95% UI: 19.87–28.13). From the NHANES study, a total of 66391 participants were included, among whom 187 had bladder cancer. The data demonstrated a significant positive association between smoking and bladder cancer risk; compared to never smokers, smokers had an adjusted odds ratio (AOR) of 2.00 (95% CI: 1.31–3.07), indicating a two-fold increased risk of bladder cancer. Further sensitivity analyses suggested that former smokers were associated with a 70% higher likelihood of risk, while current smokers showed a 265% higher likelihood. Additionally, subgroup analyses indicated differences in the observed risk across various racial groups.

**CONCLUSIONS:**

This study elucidates a significant positive association between smoking and bladder cancer risk among individuals aged ≥20 years, with notable racial/ethnic disparities observed. Our findings suggest the need for further investigation into strategies that may address these disparities. However, the cross-sectional design limits causal inference. Future longitudinal studies are warranted to investigate the carcinogenic effects of emerging tobacco products, particularly across different racial groups, to optimize prevention and control measures.

## INTRODUCTION

Bladder cancer (BCa) is one of the most common malignancies of the urinary tract. According to global health statistics, it ranks as the 10th most common cancer worldwide and exhibits high incidence and poor prognosis in several regions^1^. The primary risk factors for bladder cancer include smoking, occupational chemical exposure, and genetic predisposition. Among these risk factors, smoking is acknowledged as the most significant and modifiable contributor to bladder cancer incidence, substantially influencing both the global and national disease burden^2^.

Smoking-related bladder cancer has been extensively investigated, with multiple epidemiological studies demonstrating that tobacco use approximately doubles the risk of bladder cancer^3^. Carcinogens present in tobacco smoke, including aromatic amines and polycyclic aromatic hydrocarbons, have been demonstrated to directly damage the DNA of bladder epithelial cells. This DNA damage can induce mutations that contribute to the development and progression of bladder cancer over time^4^.

Despite the substantial burden imposed by smoking-associated bladder cancer on global healthcare systems, significant knowledge gaps remain concerning the impact of these risk factors across diverse populations, particularly in high-risk countries such as the US. Notably, although the Global Burden of Disease (GBD) study offers comprehensive data on bladder cancer risk factors, it lacks detailed insight into country-specific variations, especially for the US. This study aims to integrate the Global Burden of Disease (GBD) database with the National Health and Nutrition Examination Survey (NHANES) data to investigate the association between smoking and bladder cancer risk, focusing specifically on the US population. Additionally, the study seeks to identify potential disparities in bladder cancer prognosis, including variations in disease burden across socioeconomic groups, ethnicities, and geographical regions. Through this comprehensive analysis, the study is expected to yield valuable insights for mitigating the increasing burden of smoking-related bladder cancer in the US. The findings are anticipated to furnish a scientific basis for enhancing smoking cessation interventions and early screening strategies to improve bladder cancer prognosis among high-risk populations.

## METHODS

This study is a secondary dataset analysis based on data from the National Health and Nutrition Examination Survey (NHANES) and the Global Burden of Disease (GBD) study. The time frame for the NHANES dataset spans from 1999 to 2023, while the GBD data encompasses global health data from 1990 to 2020. The study population includes adults aged ≥20 years, and the inclusion criteria were individuals with complete data on smoking habits, bladder cancer status, and other relevant demographic and clinical variables. Participants with missing or incomplete data were excluded from the analysis.

### Global Burden of Disease (GBD) database

The Global Burden of Disease (GBD) 2021 is a comprehensive database providing anonymized data on 371 diseases, 88 risk factors, and impairments across 204 countries, territories, and five sociodemographic index (SDI) levels worldwide (40575097). The data are accessible via the Global Health Data Exchange (GHDx) platform (https://vizhub.healthdata.org/gbd-results/), which is developed and maintained by the Institute for Health Metrics and Evaluation (IHME) at the University of Washington^5^. The study involving GBD 2021 data was reviewed and approved by the Institutional Review Board (IRB) of the University of Washington, granting an exemption from the informed consent requirement^6^. This study strictly adheres to the GATHER (Guidelines for Accurate and Transparent Reporting of Global Health Estimates) guidelines to ensure the accuracy and transparency of health assessment outcomes^7^. In the GBD database, age-standardized mortality rate (ASDR) denotes the number of deaths per 100000 population across different age groups. Age-standardized disability-adjusted life years (DALYs) represent the sum of years lived with disability and years of life lost due to premature death. Total DALYs encompass both years of life lost and years lived with disability.

The estimates presented in this study, such as prevalence, DALYs, and mortality, are accompanied by both confidence intervals (CIs) and uncertainty intervals (UIs). The CIs represent the statistical uncertainty around the estimated values, based on the available data and the model used. The UIs encompass a broader scope of uncertainty, incorporating both statistical variability and model-related uncertainties, which may arise from data gaps, assumptions, or other factors inherent to the estimation process.

### National Health and Nutrition Examination Survey (NHANES) database

**Figure 1:**
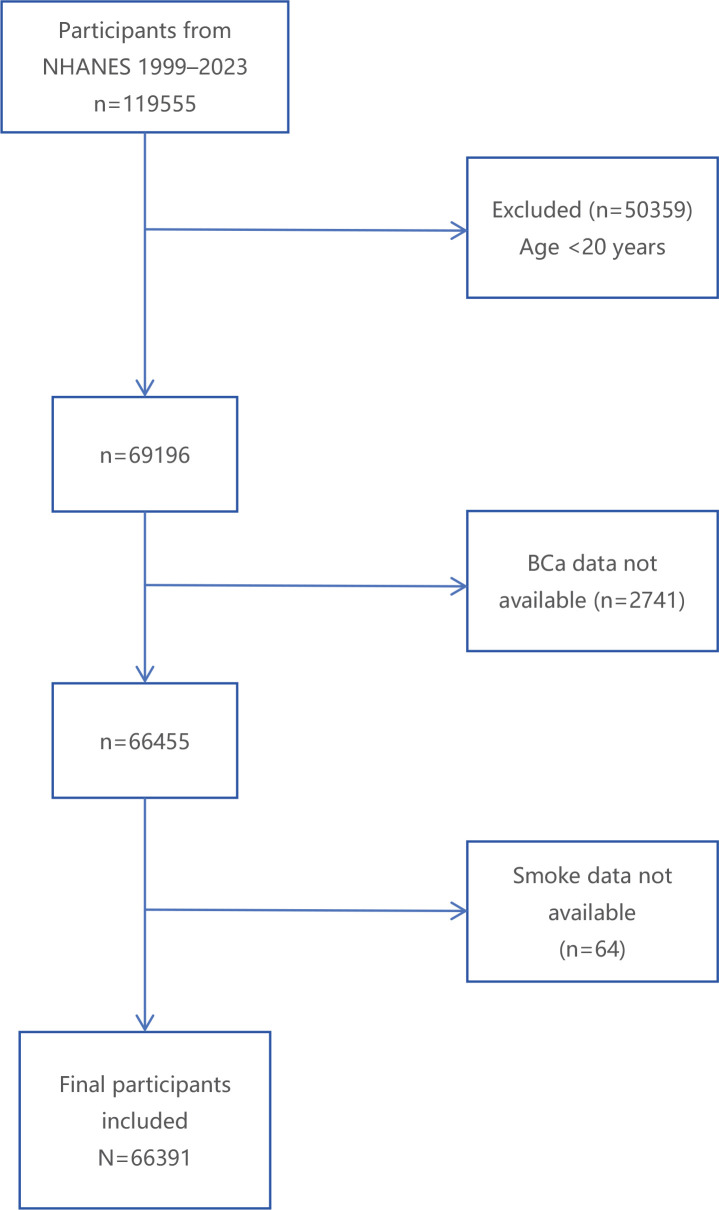
The flow chart of selection of included studies

Implemented by the National Center for Health Statistics (NCHS), the National Health and Nutrition Examination Survey (NHANES) is a stratified, multistage research program designed to assess the health and nutritional status of the US population using nationally representative data. NHANES data have been extensively utilized to investigate associations among chronic disease, nutrition, environmental exposures, and health behaviors^8^. The data collection and analysis procedures have been described in detail elsewhere^9^. A hierarchical multistage probability sampling method was employed to select representative survey respondents. The collected demographic, questionnaire, and dietary data include information on demographic characteristics, medical history, as well as dietary and lifestyle habits. Further details are available on the NHANES website (https://www.cdc.gov/nchs/nhanes/). This study analyzed subsample data from twelve NHANES survey cycles conducted between 1999 and 2023 (Figure 1). Initially, the study included 119555 NHANES participants; after applying strict inclusion and exclusion criteria, 66391 eligible participants were retained for analysis. Specifically, 50359 individuals aged <20 years, 2741 participants with missing bladder cancer-related questionnaire data, and 64 participants lacking smoking information were excluded.

### Description of variables

The primary exposure variable was a history of smoking. In NHANES, smoking data were collected via household interviews, with adults aged ≥20 years self-reporting their smoking status. Based on previous studies, participants reporting fewer than 100 cigarettes smoked over their lifetime were classified as non-smokers, whereas those who smoked 100 cigarettes or more were defined as smokers^10^. The primary outcome variable was the presence or absence of bladder cancer. According to the International Classification of Diseases (ICD), bladder cancer is coded as C67–C67.9 in ICD-10 and 188–188.9, V10.51, V16.52, and V76.3 in ICD-9^11^. Covariates were selected based on established associations with bladder cancer risk from previous literature and biological mechanisms related to bladder cancer etiology. These covariates included factors such as age, sex, race, body mass index (BMI), alcohol consumption, and comorbidities (e.g. hypertension, diabetes), which have been shown in prior studies to be associated with bladder cancer risk. Self-reported bladder cancer diagnosis information was collected using the Medical Conditions Questionnaire. Respondents were asked: ‘Has a doctor or other health professional ever informed you that you have cancer or any malignant tumor?’. If the response was ‘yes’, the type of cancer was subsequently inquired. Respondents reporting only bladder cancer (primary and solitary tumor) were defined as bladder cancer patients. Those who answered ‘no’, had other malignancies, or reported bladder cancer with a history of other cancers were classified as non-bladder cancer^12^. Former smokers were defined as individuals who had smoked more than 100 cigarettes in their lifetime but had quit, whereas current smokers were those actively smoking at the time of survey.

The NHANES database collects data via standardized household interview questionnaires and individual medical examinations. Age, sex, and ethnicity information were self-reported. Education level was categorized into two groups: lower than high school/high school or higher than high school, based on respondents’ reported highest grade completed or highest degree obtained. Marital status was classified as married/partnered, divorced/widowed/separated, or never married. The poverty income ratio (PIR) represents the ratio of a household’s self-reported income to the federal poverty threshold. Body mass index (BMI) was calculated as weight in kg divided by the square of height in meters (kg/m²). Alcohol consumption was defined as the consumption of at least 12 drinks within one year prior to the survey. Hypertension was defined as a history of hypertension or measured systolic blood pressure ≥140 mmHg or diastolic blood pressure ≥90 mmHg. Diabetes mellitus was defined as a history of diabetes, use of insulin or glucose-lowering medications, glycosylated hemoglobin ≥6.5%, fasting blood glucose ≥126 mg/dL, or 2-hour postprandial blood glucose ≥200 mg/dL. Each participant completed a physical activity questionnaire addressing all physical activity performed in the preceding 30 days. Based on adherence to national physical activity guidelines, participants were categorized into low physical activity (<500 MET-minutes/week) and high physical activity (≥500 MET-minutes/week) groups^13^. To ensure data quality, a comprehensive evaluation of missing data was conducted. Variables with missing rates exceeding 10% were addressed using multiple imputation.

This study is a secondary dataset analysis based on cross-sectional data, reported in accordance with the STROCSS guidelines^14^. Anonymized data from public databases, including the Global Burden of Disease (GBD) and National Health and Nutrition Examination Survey (NHANES), were utilized in this study. Ethical approval was obtained in the original studies; therefore, no additional ethical approval was required for the present analysis.

### Statistical analysis

Using the 2021 data from the United Nations standard projections data set, age-standardized rates (ASR) such as age-standardized death rate (ASDR), and age-standardized disability-adjusted life years rate (ASDAR), were computed. The rates were derived based on the formula:


$$\text{ASR}=\frac{\overset{A}{\underset{i=1}{\sum }}a_{i}W_{i}}{\overset{A}{\underset{i=1}{\sum }}W_{i}}\times 100000$$

where A denotes the number of age groups, i denotes the ith age group, a_i_ is the rate to be standardized, and w_i_ is the number of standard populations in the same age group. Comparing age-standardized mortality rate (ASDR) and age-standardized disability-adjusted life year rate (ASDAR) between the US and global populations requires careful consideration. While we have provided descriptive comparisons between the two regions, we recognize that formal statistical tests to assess differences between the US and global ASDR and ASDAR may not be appropriate. The Global Burden of Disease (GBD) database spans multiple years and includes variations in data collection methods, statistical estimation techniques, and reporting conventions across different countries and regions. These differences could influence the accuracy and comparability of the data. Therefore, rather than conducting formal statistical comparisons, our analysis focuses on providing an overview of the trends and disparities observed in the US and global indices, acknowledging the limitations of cross-sectional data from different time periods. This distinction will be emphasized in the revised manuscript to clarify the scope and interpretation of the results.

This study utilized the 2021 Global Burden of Disease (GBD) database to analyze risk factors associated with the bladder cancer burden and to evaluate the attributable deaths and disability-adjusted life years (DALYs) for each risk factor. Additionally, this study focuses on comparing the number of attributable deaths and the magnitude of attributable DALYs between the US and the global region. All statistical tests were two-tailed, and statistical significance was defined as a p<0.05.

This study examined the relationship between smoking and the incidence of bladder cancer based on data from the National Health and Nutrition Examination Survey (NHANES). Continuous variables were presented as mean ± standard error, while categorical variables were expressed as frequency (percentage). Comparisons between groups were conducted using t-tests, Mann–Whitney U tests, or chi-squared (χ^2^) tests. Multivariable logistic regression models were employed to assess the association between smoking and bladder cancer.

Three hierarchical adjustment models were constructed: Model 1 (unadjusted); Model 2 adjusted for age, sex, and race; and Model 3 adjusted as for Model 2 plus education level, marital status, drinking, physical activity, BMI, PIR, hypertension and diabetes. Results were reported as odds ratios (ORs) with 95% confidence intervals (CIs). Stratified analyses across subgroups were conducted along with interaction tests to evaluate the consistency of the association between smoking and bladder cancer. Subgroup findings were visualized through forest plots. To account for the complex, multistage probability sampling design of NHANES, survey weights were incorporated throughout the analyses to ensure nationally representative estimates.

## RESULTS

### Global and US bladder cancer burden and smoking as major risk factors

**Figure 2:**
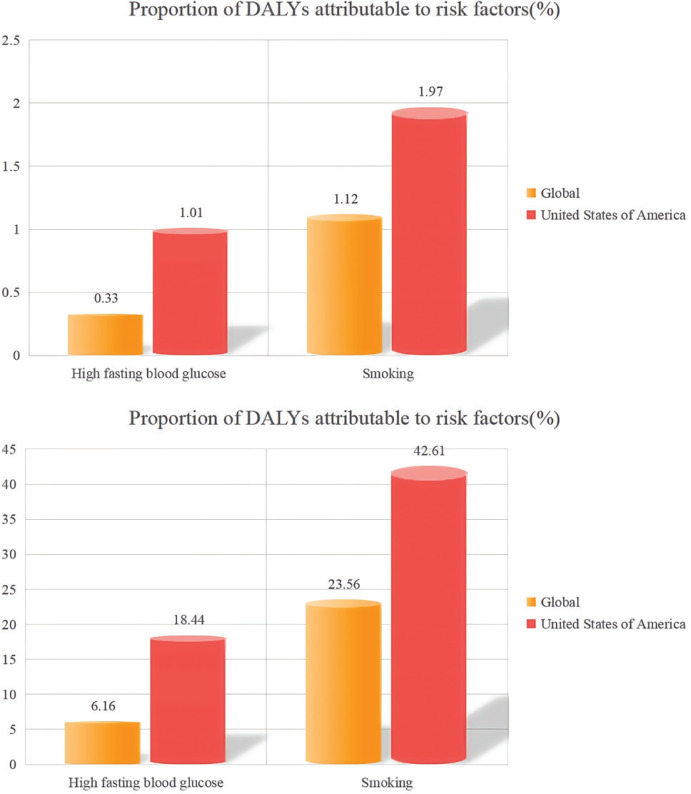
Comparative analysis of smoking-attributable bladder cancer burden between global and US populations in 2021

Figure 2 and Table 1 present a detailed analysis of risk factors contributing to the burden of bladder cancer, emphasizing the impact of smoking both globally and in the US. In 2021, the global number of deaths from smoking-related bladder cancer was 58766.87 (95% UI: 49381.34–70891.72), and the number of disability-adjusted life years (DALYs) attributable to smoking-related bladder cancer was 107082.78 (95% UI: 87698.70–129617.13). During the same period, the number of deaths from bladder cancer caused by smoking in the US was 2535.77 (95% UI: 334.76–5692.50), and the corresponding number of DALYs was 1238303.16 (95% UI: 1044303.32–1478220.98). Notably, the age-standardized mortality rate (ASDR) in the US was 1.97 (95% UI: 1.57–2.47), which was significantly higher than the global rate of 1.12 (95% UI: 0.94–1.35). The age-standardized disability-adjusted life year (ASDAR) rate in the US was also markedly higher than the global average, with rates of 42.60 (95% UI: 34.89–51.57) and 23.56 (95% UI: 19.87–28.13), respectively.

**Table 1:** Global and US bladder cancer burden in 2021

*Burden*	Prevalence (95% UI/CI*)
**Global**	
Prevalence number	3023314.90 (3221739.72–2821046.10)
Age standardized prevalence rate	57.53 (61.30–53.68)
DALYs number	4392124.72 (4808322.14–4059076.05)
Age-standardized DALYs rate	83.57 (91.49–77.23)
Deaths number	221822.93 (242262.50–200505.25)
Age-standardized mortality	4.22 (4.61–3.82)
**US**	
Prevalence number	585276.99 (609652.36–549975.05)
Age standardized prevalence rate	232.87 (242.57–218.82)
DALYs number	407070.28 (433038.16–371610.62)
Age-standardized DALYs rate	161.97 (172.30–147.86)
Deaths number	21496.51 (22906.35–19022.05)
Age-standardized mortality	8.55 (9.11–7.57)

* 95% Confidence interval (CI) represents statistical precision, indicating the range within which the true value is expected to lie with 95% confidence based on the available data. 95% Uncertainty interval (UI) reflects broader uncertainty, incorporating not only statistical variability but also model assumptions and potential data limitations. For further details, please refer to the Methods section.

**Figure 3:**
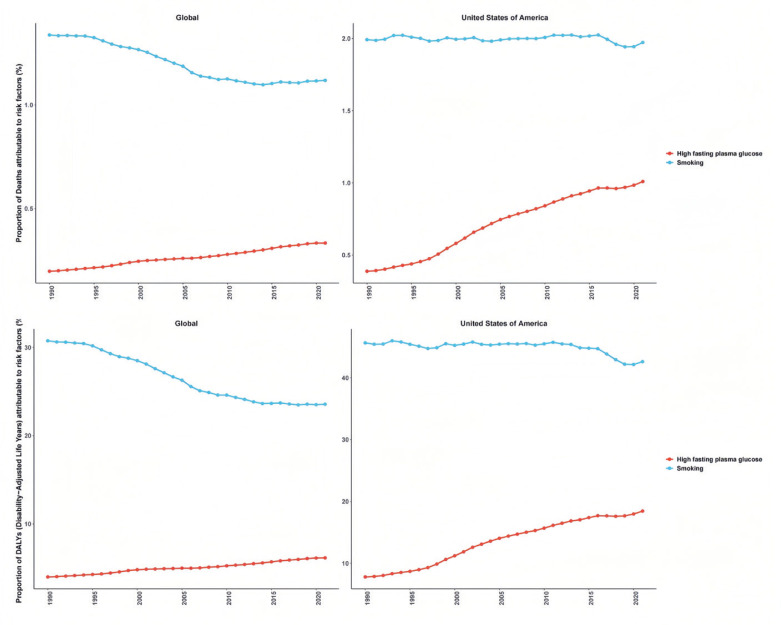
Temporal trends in smoking-related bladder cancer burden in 1990–2021

The temporal trend analysis (Figure 3) indicates that the burden of bladder cancer attributable to smoking has generally declined on a global scale, whereas no significant reduction has been observed in the US. Between 1990 and 2021, the number of deaths from smoking-related bladder cancer, as well as the associated disability-adjusted life years (DALYs), increased both globally and in the US. While global indicators demonstrate a decline in age-standardized death rates (ASDR) and age-standardized DALY rates (ASDAR), an opposite trend has emerged in the US, reflecting an increasing burden of smoking-related bladder cancer. In particular, over the past decade, ASDAR in the US exhibited a modest decline; however, since 2020, it has shown an upward trajectory.

**Figure 4:**
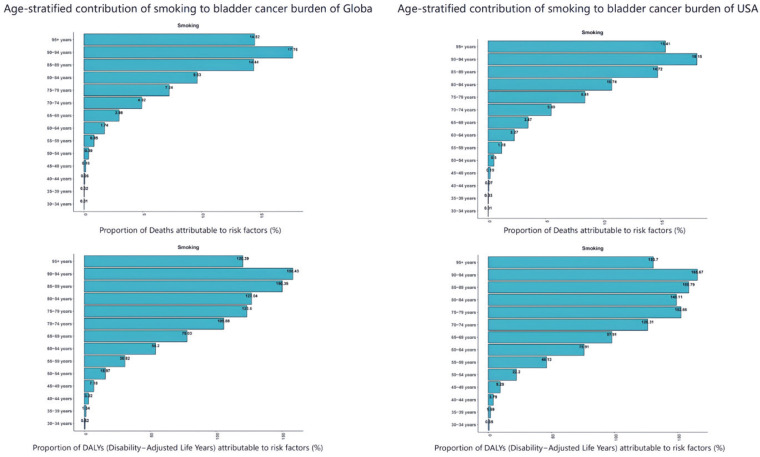
Age-stratified contribution of smoking to bladder cancer burden

The age-standardized death rate (ASDR) and age-standardized disability-adjusted life year rate (ASDAR) for smoking-related bladder cancer were highest in the 90–94 years age group, both globally and in the US, underscoring the cumulative and long-term health impacts of smoking (Figure 4). For the contribution of different risk factors by age group to bladder cancer deaths, with smoking identified as the predominant contributor both globally and in the US. Smoking-attributable bladder cancer mortality reaches its peak at 70–74 years of age worldwide and at 75–79 years in the US. DALYs attributable to smoking peaked in the 70–74 years age range in both global and US populations.

### NHANES study participant characteristics

A total of 119555 participants from NHANES were included in this study. After excluding individuals with missing data on bladder cancer status or smoking history, the final analytic sample comprised 66391 participants, including 66204 without bladder cancer and 187 with bladder cancer. Table 2 presents the demographic characteristics stratified by bladder cancer status. Significant differences were observed between the bladder cancer and non-bladder cancer groups in terms of smoking history, age, sex, ethnicity, marital status, physical activity, and the prevalence of hypertension and diabetes, whereas no significant differences were noted in BMI, education level, or alcohol consumption. Patients with bladder cancer had a higher proportion of males, an older average age, and were more likely to have a history of smoking, low physical activity, as well as comorbid hypertension and diabetes.

**Table 2:** Baseline characteristics of participants aged ≥20 years included in the NHANES 1999–2023 dataset, stratified by bladder cancer (BCa) status

*Characteristics*	*Non-BCa (N=66204)* *n (%)*	*BCa (N=187)* *n (%)*	*p*
**Age** (years), mean (SE)	47.23 (0.16)	72.20 (1.00)	**<0.001**
BMI (kg/m^2^), mean (SE)	28.88 (0.06)	29.13 (0.52)	0.635
**Sex**			**<0.001**
Male	31524 (48.04)	140 (70.96)	
Female	34680 (51.96)	47 (29.04)	
**Race**			**<0.001**
Mexican American	10427 (8.08)	10 (1.72)	
Other Hispanic	5701 (6.16)	5 (0.93)	
Non-Hispanic White	29935 (66.83)	147 (87.81)	
Non-Hispanic Black	13624 (11.28)	14 (3.85)	
Other race	6517 (7.64)	11 (5.69)	
**Education level**			0.661
Lower than high school/high school	16746 (16.46)	52 (17.66)	
Higher than high school	49458 (83.54)	135 (82.34)	
**Marital status**			**0.014**
Married/living with partner	38843 (62.87)	125 (68.19)	
Widowed/divorced/separated	18752 (23.68)	55 (27.88)	
Never married	8609 (13.44)	7 (3.92)	
**Smoke**			**<0.001**
No	36594 (54.84)	54 (29.68)	
Yes	29610 (45.16)	133 (70.32)	
**Drinking**			0.511
No	19237 (25.11)	47 (22.42)	
Yes	46967 (74.89)	140 (77.58)	
**Physical activity**			**0.017**
No	25277 (34.46)	86 (44.24)	
Yes	40927 (65.54)	101 (55.76)	
**Hypertension**			**<0.001**
No	38395 (63.57)	63 (37.64)	
Yes	27809 (36.43)	124 (62.36)	
**Diabetes**			**<0.001**
No	54684 (86.93)	130 (72.49)	
Yes	11520 (13.07)	57 (27.51)	
**PIR**			0.198
<1.3	20151 (21.91)	43 (16.79)	
1.3–3.5	24927 (35.77)	85 (42.69)	
>3.5	21126 (42.32)	59 (40.52)	

Statistical analyses were performed using R 4.3.0. Continuous data with a normal distribution are expressed as mean (standard error, SE), and comparisons between groups were made using an independent two-sample t-test. Categorical data are presented as n (%), and group comparisons were performed using the chi-squared test or Fisher’s exact test, depending on the distribution of the data. PIR: poverty income ratio. A p<0.05 was considered statistically significant.

### Association analysis between smoking and bladder cancer

To explore the relationship between smoking history and the risk of bladder cancer, three multivariable logistic regression models were constructed (Table 3). All models demonstrated a statistically significant positive association between smoking and the incidence of bladder cancer. The odds ratios (ORs) and 95% confidence intervals (CIs) for Models 1 (crude ORs), 2 (adjusted ORs), and 3 (adjusted ORs) were 2.88 (95% CI: 1.93–4.29), 2.01 (95% CI: 1.31–3.07), and 2.00 (95% CI: 1.31–3.07), respectively. The fully adjusted model (Model 3) indicated that smokers had approximately twice the risk of developing bladder cancer compared to non-smokers.

**Table 3:** Association analysis between smoking and bladder cancer

*Smoking status*	*Model 1*		*Model 2*		*Model 3*	
	*OR (95% CI)*	*p*	*AOR (95% CI)*	*p*	*AOR (95% CI)*	*p*
Non-smokers ®	1.00		1.00		1.00	
Smokers	2.88 (1.93–4.29)	**<0.001**	2.01 (1.31–3.07)	**0.002**	2.00 (1.31–3.07)	**0.002**
p for trend	**<0.001**		**<0.001**		**<0.001**	

Model 1: no covariates were adjusted. AOR: adjusted odds ratio. Model 2: adjusted for sex, age and race. Model 3: adjusted as for Model 2 plus education level, marital status, drinking, physical activity, BMI, PIR, hypertension and diabetes. ® Reference category.

### Subgroup analysis

**Figure 5:**
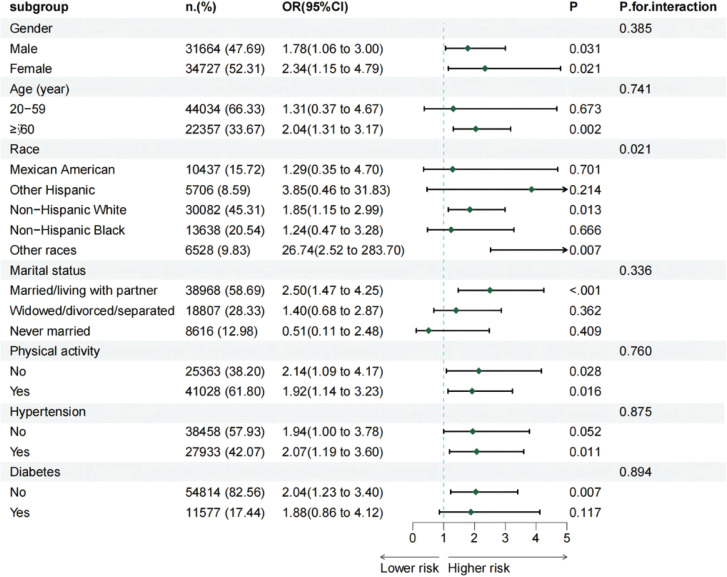
Subgroup heterogeneity in smoking-associated bladder cancer risk

To assess the robustness of the association between smoking history and bladder cancer across population subgroups, stratified analyses and interaction tests were conducted by sex, age, ethnicity, education level, marital status, alcohol consumption, physical activity, and the presence of hypertension or diabetes. The results (Figure 5) indicated that ethnicity was the only variable that significantly modified the association between smoking and bladder cancer (interaction p=0.021).

### Sensitivity analysis

To verify the robustness of the findings, a sensitivity analysis was conducted by further categorizing NHANES participants with a smoking history into current and former smokers. As shown in Table 4, the risk of bladder cancer was significantly elevated in both current and former smokers across all models (p for trend <0.05). In the fully adjusted model, the risk of bladder cancer was 70% higher in former smokers (OR=1.70; 95% CI: 1.08–2.68) and 265% higher in current smokers (OR=3.65; 95% CI: 2.02–6.59).

**Table 4:** Sensitivity analyses of smoking–bladder cancer association

Smoking status	Model 1		Model 2		Model 3	
	OR (95% CI)	p	AOR (95% CI)	p	AOR (95% CI)	p
Non-smokers ®	1.00		1.00		1.00	
Former smokers	3.77 (2.44–5.83)	**<0.001**	1.71 (1.09–2.70)	**0.022**	1.70 (1.08–2.68)	**0.024**
Current smokers	1.81 (1.06–3.10)	**0.031**	3.36 (1.90–5.94)	**<0.001**	3.65 (2.02–6.59)	**<0.001**
p for trend	**<0.001**		**<0.001**		**<0.001**	

Model 1: no covariates were adjusted. Model 2: adjusted for sex, age and race. Model 3: adjusted as for Model 2 plus education level, marital status, drinking, physical activity, BMI, PIR, hypertension and diabetes. ® Reference category.

## DISCUSSION

This study evaluated the impact of smoking on bladder cancer risk and disease burden by integrating cross-national macro-level data from the Global Burden of Disease (GBD) study and nationally representative individual-level data from NHANES in the US. Although global trends since 1990 have shown a decline in smoking-attributed age-standardized death rates (ASDR) and disability-adjusted life year rates (ASDAR) for bladder cancer, our analysis demonstrates a persistent upward trend in the US, indicating substantial challenges in bladder cancer prevention and control efforts. Data from the 2021 GBD study indicated that smoking was the leading risk factor for bladder cancer mortality and DALYs in the US. Analysis of NHANES individual-level data further confirmed a significant association between smoking history and bladder cancer risk. In the fully adjusted model, smokers exhibited a 100% higher risk of developing bladder cancer compared to non-smokers. Sensitivity analysis revealed a 70% increased risk among former smokers and a 265% increase among current smokers, relative to non-smokers. This finding not only quantifies the differential impact of smoking on the burden of bladder cancer globally and in the US, but also emphasizes the importance of tobacco control as a critical intervention strategy, providing a scientific basis for formulating and optimizing targeted bladder cancer prevention policies.

The rising burden of smoking-related bladder cancer in the US may be attributed to several interrelated factors. First, there are significant regional and population-level disparities in tobacco use patterns and tobacco control policies. Although tobacco control efforts over the past five decades have successfully reduced overall smoking prevalence, the health benefits have been unevenly distributed across groups stratified by socioeconomic status (SES), race/ethnicity, and sexual orientation, contributing to persistent disparities in health outcomes. For example, a 10-year observational study found that smoking rates were nearly three times higher among individuals with low SES compared to those with high SES PMID^15^. In addition, the widespread use of new tobacco products, particularly e-cigarettes, has contributed to the rising burden of bladder cancer in the US. According to the U.S. Centers for Disease Control and Prevention, e-cigarette use continues to increase, and e-cigarettes along with other tobacco products remain major contributors to preventable morbidity and mortality in the country, despite the decline in conventional cigarette smoking^16^. E-cigarette vapor and liquids contain a range of toxic substances, including aldehydes, heavy metals, and polycyclic aromatic hydrocarbons (PAHs), all of which possess carcinogenic potential^17-19^. Furthermore, disparities in access to and quality of healthcare services among different populations exacerbate regional and population-level differences in the burden of smoking-related bladder cancer. Economic disadvantages significantly hinder access to early diagnosis and optimal treatment^20^, particularly in low-resource communities where high smoking prevalence and limited cessation resources contribute to poorer clinical outcomes^21^.

This study confirmed the role of smoking as an independent risk factor for bladder cancer in a large, nationally representative sample of the US, in line with prior epidemiological evidence. Early case–control studies observed that smokers had approximately twice the risk of developing bladder cancer compared to non-smokers, and a large prospective cohort study in Australia further reported that smoking accounted for nearly 50% of the total bladder cancer burden. Molecular studies have demonstrated that tobacco combustion products contain potent carcinogens, including polycyclic aromatic hydrocarbons (PAHs) and aromatic amines, which reach the bladder epithelium at high concentrations via urinary excretion, thereby inducing DNA damage^22^. In vitro experiments have shown that tobacco smoke extract activates the MAPK/AP-1 signaling pathway and promotes aberrant proliferation of urothelial cells^23^, while nicotine contributes to tumor progression and chemoresistance through activation of the PI3K/AKT/mTOR signaling axis^24^.

In conclusion, the escalating burden of smoking-associated bladder cancer in the US highlights the urgent need for strengthened tobacco control efforts, particularly among populations with low socioeconomic status (SES) and racial or ethnic minorities. This study provides scientific evidence to inform multi-dimensional and targeted public health interventions aimed at reducing tobacco-related cancer risk. These include intensifying conventional tobacco control measures (e.g. cigarette taxation, smoke-free legislation, and public media campaigns) while expanding access to smoking cessation services in underserved populations. In addition, stronger regulation of emerging tobacco products is needed to limit exposure to harmful constituents, with an emphasis on protecting youth. Integrating smoking cessation interventions into primary care systems and urologic oncology care pathways may further address disparities in diagnosis and treatment. The implementation of comprehensive, equity-oriented tobacco control strategies may help reverse the concerning upward trajectory of bladder cancer burden in the US.

### Limitations

Several limitations should be acknowledged. First, although GBD data are extensive, their quality and completeness vary across countries, and estimation uncertainty remains high in low sociodemographic index (SDI) regions due to limited data availability. Second, the relatively small number of bladder cancer cases in the NHANES sample reduced the statistical power for subgroup analyses. Additionally, self-reporting bias and residual confounding due to unmeasured variables may have influenced the interpretation of the results. The findings are primarily applicable to individuals aged ≥20 years in the US, thus limiting the generalizability of the results to other age groups or populations. Future studies should further investigate the long-term carcinogenic risk and socioeconomic impact of emerging tobacco products using longitudinal study designs and multicenter cancer registry databases.

## CONCLUSIONS

This study is the first to integrate population-level data from the Global Burden of Disease (GBD) study with individual-level data from NHANES to systematically explore the association between smoking and the increasing burden of bladder cancer in the US. The results suggest that smokers have a two-fold increased likelihood of developing bladder cancer compared to non-smokers. However, given the observational nature of the data, causal inference cannot be drawn from these findings. These results provide valuable insights for enhancing bladder cancer screening and implementing targeted tobacco control strategies among high-risk populations. Future studies should investigate the long-term carcinogenic risks of emerging tobacco products, such as e-cigarettes, through the integration of cancer registry data and mechanistic studies, to inform improved bladder cancer prevention and control strategies.

## Supplementary Material



## Data Availability

The data sets generated and/or analyzed during the current study are available in the GBD repository (http://ghdx.healthdata.org/gbd-results-tool). The original contributions presented in the study are included in the Supplementary file. Further inquiries can be directed to the corresponding author.

## References

[1] Park JH, Hong JY, Han K, Shen JJ. Sex-specific associations of glycemic status and smoking with bladder cancer risk: a nationwide cohort study. Cancers (Basel). 2025;17(13):2262. doi:10.3390/cancers17132262 40647559 PMC12249375

[2] Yuan J, Chen L, Zhou J, et al. Global burden of bladder cancer attributable to smoking in 204 countries and territories, 1990-2019. Heliyon. 2024;10(13):e34114. doi:10.1016/j.heliyon.2024.e34114 39091950 PMC11292503

[3] Dyrskjøt L, Hansel DE, Efstathiou JA, et al. Bladder cancer. Nat Rev Dis Primers. 2023;9(1):58. doi:10.1038/s41572-023-00468-9 37884563 PMC11218610

[4] Stämpfli MR, Anderson GP. How cigarette smoke skews immune responses to promote infection, lung disease and cancer. Nat Rev Immunol. 2009;9(5):377-384. doi:10.1038/nri2530 19330016

[5] Leung DK, Wong CH, Ko IC, et al. Global trends in the incidence, mortality, and risk-attributable deaths for prostate, bladder, and kidney cancers: a systematic analysis from the Global Burden of Disease Study 2021. Eur Urol Oncol. doi:10.1016/j.euo.2025.05.007 40441940

[6] Qi K, Cheng H, Jiang Y, Zheng Y. Contribution of smoking to the global burden of bladder cancer from 1990 to 2021 and projections to 2046. Tob Induc Dis. 2025;23(March):44. doi:10.18332/tid/202237 PMC11951971 40161903

[7] Zi H, Liu MY, Luo LS, et al. Global burden of benign prostatic hyperplasia, urinary tract infections, urolithiasis, bladder cancer, kidney cancer, and prostate cancer from 1990 to 2021. Mil Med Res. 2024;11(1):64. doi:10.1186/s40779-024-00569-w 39294748 PMC11409598

[8] Huang M, Li H, Chen J, et al. Blood lead levels and bladder cancer among US participants: NHANES 1999-2018. BMC Public Health. 2025;25(1):416. doi:10.1186/s12889-025-21549-2 39894828 PMC11787758

[9] Gao X, Qi J, Du B, Weng X, Lai J, Wu R. Combined influence of nutritional and inflammatory status and breast cancer: findings from the NHANES. BMC Public Health. 2024;24(1):2245. doi:10.1186/s12889-024-19727-9 39160507 PMC11331661

[10] Hou W, Chen S, Zhu C, Gu Y, Zhu L, Zhou Z. Associations between smoke exposure and osteoporosis or osteopenia in a US NHANES population of elderly individuals. Front Endocrinol (Lausanne). 2023;14:1074574. doi:10.3389/fendo.2023.1074574 36817605 PMC9935577

[11] Chen X, Guo H, Cao S, et al. Disease burden of bladder cancer in China and the different SDI regions over the world from 1990 to 2021. Sci Rep. 2025;15(1):20811. doi:10.1038/s41598-025-08634-7 40596449 PMC12219441

[12] Teng C, Lu W, Che J, Wu Y, Meng D, Shan Y. Association of pro-inflammatory diet, smoking, and alcohol consumption with bladder cancer: evidence from case-control and NHANES studies from 1999 to 2020. Nutrients. 2024;16(11):1793. doi:10.3390/nu16111793 38892724 PMC11174752

[13] MacGregor KA, Gallagher IJ, Moran CN. Relationship between insulin sensitivity and menstrual cycle is modified by BMI, fitness, and physical activity in NHANES. J Clin Endocrinol Metab. 2021;106(10):2979-2990. doi:10.1210/clinem/dgab415 34111293 PMC8475204

[14] Mathew G, Agha R; STROCSS Group. STROCSS 2021: strengthening the reporting of cohort, cross-sectional and case-control studies in surgery. Ann Med Surg (Lond). 2021;72:103026. doi:10.1016/j.amsu.2021.103026 34820121 PMC8599107

[15] Jamal A, King BA, Neff LJ, Whitmill J, Babb SD, Graffunder CM. Current cigarette smoking among adults - United States, 2005-2015. MMWR Morb Mortal Wkly Rep. 2016;65(44):1205-1211. doi:10.15585/mmwr.mm6544a2 27832052

[16] Cornelius ME, Loretan CG, Jamal A, et al. Tobacco product use among adults - United States, 2021. MMWR Morb Mortal Wkly Rep. 2023;72(18):475-483. doi:10.15585/mmwr.mm7218a1 37141154 PMC10168602

[17] Oni TM, Sadhasivam B, Floyd EL. Assessment of vape shop built environment: airborne nicotine, particulate matter, ventilation, hazard identification, workplace practices, and safety perceptions. Ann Work Expo Health. 2025;69(5):510-519. doi:10.1093/annweh/wxaf018 40577798 PMC12208366

[18] U.S. Department of Health and Human Services. Public Health Service. Agency for Toxic Substances and Disease Registry (ATSDR). Toxicological Profile for Polycyclic Aromatic Hydrocarbons; 1995. Accessed November 09, 2025. https://www.atsdr.cdc.gov/toxprofiles/tp69.pdf 38091452

[19] Besaratinia A. Electronic cigarette-derived metals: exposure and health risks in vapers. Chem Res Toxicol. 2025;38(4):542-556. doi:10.1021/acs.chemrestox.4c00520 40094421 PMC12136401

[20] Walter AW, Lee JW, Streck JM, et al. The effect of neighborhood socioeconomic disadvantage on smoking status, quit attempts, and receipt of cessation support among adults with cancer: results from nine ECOG-ACRIN cancer research group trials. Cancer. 2024;130(3):439-452. doi:10.1002/cncr.35039 37795845 PMC10841845

[21] Valiente R, Escobar F, Urtasun M, Franco M, Shortt NK, Sureda X. Tobacco retail environment and smoking: a systematic review of geographic exposure measures and implications for future studies. Nicotine Tob Res. 2021;23(8):1263-1273. doi:10.1093/ntr/ntaa223 33155040

[22] Zhao X, Wang Y, Liang C. Cigarette smoking and risk of bladder cancer: a dose-response meta-analysis. Int Urol Nephrol. 2022;54(6):1169-1185. doi:10.1007/s11255-022-03173-w 35332429

[23] Geng H, Zhao L, Liang Z, et al. Cigarette smoke extract-induced proliferation of normal human urothelial cells via the MAPK/AP-1 pathway. Oncol Lett. 2017;13(1):469-475. doi:10.3892/ol.2016.5407 28123584 PMC5245078

[24] Yuge K, Kikuchi E, Hagiwara M, et al. Nicotine induces tumor growth and chemoresistance through activation of the PI3K/Akt/mTOR pathway in bladder cancer. Mol Cancer Ther. 2015;14(9):2112-2120. doi:10.1158/1535-7163.MCT-15-0140 26184482

